# Activation by NarL at the *Escherichia coli ogt* promoter

**DOI:** 10.1042/BCJ20200408

**Published:** 2020-08-07

**Authors:** Patcharawarin Ruanto, David L. Chismon, Joanne Hothersall, Rita E. Godfrey, David J. Lee, Stephen J. W. Busby, Douglas F. Browning

**Affiliations:** 1 Institute of Microbiology and Infection and School of Biosciences, University of Birmingham, Edgbaston, Birmingham B15 2TT, U.K.; 2 Department of Life Sciences, School of Health Sciences, Birmingham City University, Birmingham B15 3TN, U.K.

**Keywords:** molecular interactions, NarL, promoters, transcription activation

## Abstract

The *Escherichia coli* NarX/NarL two-component response-regulator system regulates gene expression in response to nitrate ions and the NarL protein is a global transcription factor, which activates transcript initiation at many target promoters. One such target, the *E. coli ogt* promoter, which controls the expression of an O^6^-alkylguanine-DNA-alkyltransferase, is dependent on NarL binding to two DNA targets centred at positions −44.5 and −77.5 upstream from the transcript start. Here, we describe *ogt* promoter derivatives that can be activated solely by NarL binding either at position −44.5 or position −77.5. We show that NarL can also activate the *ogt* promoter when located at position −67.5. We present data to argue that NarL-dependent activation of transcript initiation at the *ogt* promoter results from a direct interaction between NarL and a determinant in the C-terminal domain of the RNA polymerase α subunit. Footprinting experiments show that, at the −44.5 promoter, NarL and the C-terminal domain of the RNA polymerase α subunit bind to opposite faces of promoter DNA, suggesting an unusual mechanism of transcription activation. Our work suggests new organisations for activator-dependent transcription at promoters and future applications for biotechnology.

## Introduction

The first steps in the pathway to transcription initiation at bacterial promoters involve the recognition of different promoter elements by the multisubunit bacterial DNA-dependent RNA polymerase holoenzyme (RNAP). Thus, for example, a DNA-binding determinant in Domain 4 of the RNAP σ subunit interacts with the promoter ‘−35 element’ (a hexamer base sequence located 35 bp upstream from the transcript start), whilst the C-terminal domain of the RNAP α subunit (αCTD) interacts with the promoter UP element (a short AT-rich DNA segment located immediately upstream of the −35 element) [1]. However, at many bacterial promoters, these interactions are not possible without the intervention of a transcription factor. These are mostly sequence-specific DNA binding proteins whose activity is triggered by a specific signal, and this couples the transcription of specific genes to particular environmental cues [2]. Many of these factors function by making one or more direct contacts with RNAP that recruit it to a target promoter, thereby activating transcription [3]. Some activators interact with Domain 4 of the RNAP σ subunit, and hence must bind adjacent to the target promoter −35 element, whilst others contact αCTD and bind further upstream [1,3]. Most bacterial transcription factors are homodimers. At some promoters, the binding of just one dimer is sufficient for full activation, whereas at others, two dimers are required, and this provides a simple mechanism whereby the activity of a promoter can be coupled to different activators [3,4]. Although bacterial transcription activators have been studied for over 50 years, interest in them is sustained, as they continue to provide new insights into microbial life and its adaptations, but also, many activators and their target promoters have been adopted as components in the construction of new genetic circuits for synthetic biology applications [5].

**Figure 1.**
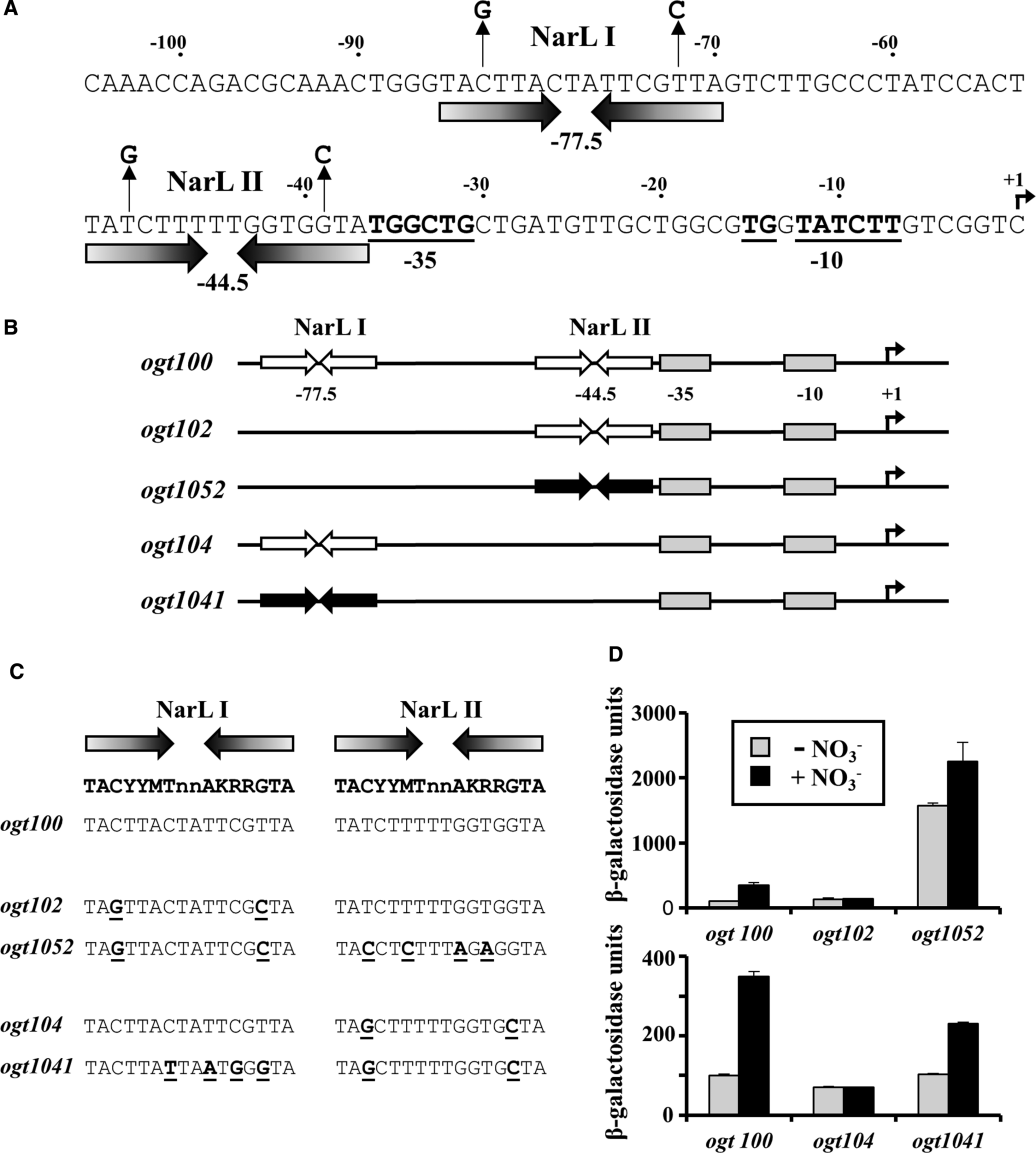
Activities of the *ogt* promoter derivatives used in this study. (**A**) The panel shows the base sequence of the *E. coli* K-12 *ogt* promoter fragment from position −105 to +1 relative to the transcript start site (+1), which is indicated by a bent arrow. Inverted arrows show the two DNA sites for NarL and the centre of each site is indicated. The −10, extended −10 and −35 elements are underlined and in bold. (**B**) A schematic representation of the *ogt100*, *ogt102*, *ogt1052*, *ogt104* and *ogt1041* promoter fragments used in this study. The NarL binding sites are shown as inverted arrows, −35 and −10 promoter elements are shown as boxes and the transcript start site (+1), is indicated by a bent arrow. Improved NarL binding sites are shown as filled black arrows. (**C**) The panel shows base sequences of the DNA sites for NarL in the *ogt100*, *ogt102*, *ogt1052, ogt104* and *ogt1041* promoter fragments. The NarL 7-2-7 consensus binding sequence is shown in boldface above (Y = C/T, M = A/C, K = G/T, R = A/G) [23]. The underlined sequences indicate the bases that have been changed in each promoter fragment. (**D**) The panel shows β-galactosidase activities measured in wild type *E. coli* K-12 JCB387 cells, carrying the *ogt100*, *ogt102, ogt1052, ogt104* and *ogt1041* promoters as *lacZ* fusions, cloned into expression vector pRW50 [17]. Cells were grown in minimal salts media supplemented with 20 mM sodium nitrate, where indicated. β-galactosidase activities are expressed as nmol ONPG hydrolysed min^−1^ mg^−1^ dry cell mass and represent the average of three independent experiments.

Two-component systems (consisting of a sensor-kinase protein that acts on a response-regulator protein) provide a way for bacterial cells to tailor their gene expression to external stimuli, and the *Escherichia coli* NarL protein is a typical two-component system response-regulator [6,7]. NarL can act as a transcription activator or repressor, and its activity is induced by phosphorylation by two inner membrane-bound sensor kinases, NarX and NarQ, that are activated by nitrate or nitrite ions in the periplasmic space [8,9]. Once phosphorylated, NarL binds to specific DNA sites at target promoters to influence gene expression. Many of these sites consist of two copies of a 7-base element, organised as an inverted repeat, separated by 2 bp (known as the ‘7-2-7’ sequence), that accommodates the binding of dimeric NarL [10]. The Regulon DB database [11] for transcription regulation in *E. coli* lists 26 gene regulatory regions where NarL has a direct effect on transcript initiation, and it functions as an activator at 11 of these. Most of these regulatory regions are quite complex, involving several transcription factors, as well as nucleoid-associated proteins (NAPs), with NarL binding at one or more 7-2-7 sequences, and sometimes single 7-base elements [7,11]. Recall that the primary role of NAPs is the compaction of the bacterial chromosomal DNA into the nucleoid, but this often results in suppression of promoter activity [12]. Hence, we previously showed that, at some NarL-activated promoters, the primary function of NarL is not to recruit RNAP but, rather, to reorganise the NAPs to permit transcription initiation [13]. However, inspection of the available data identified two relatively simple NarL-dependent promoters, where NarL alone is sufficient for full induction [14,15]. At one, the *yeaR*-*yoaG* promoter, NarL binding to a single 7-2-7 sequence, located just upstream of the promoter −35 element, is sufficient for activation [14]. At the other, the *ogt* promoter, activation requires NarL binding to tandem 7-2-7 operator sites, with one abutting the promoter −35 element and the other located 43 bp upstream (Figure 1A) [15]. In this work, our aim was to understand NarL-dependent activation at the *ogt* promoter and to investigate possible interactions between NarL and RNAP.

## Materials and methods

### Bacterial strains, plasmids and promoter fragments

*E. coli K-12* bacterial strains and plasmids used in this study are listed in Supplementary Table S1 and oligonucleotide primers are in Supplementary Table S2. The *ogt* promoter derivatives, *ogt100*, *ogt102* and *ogt104*, cloned into plasmid pSR and into the *lacZ* expression vector pRW50, were reported previously [15–17]. The *ogt1052* promoter was constructed by a two-step megaprimer PCR method [18]. Primers ogt105 and pSRDown were used to amplify a megaprimer from the pSR/*ogt100* template. The purified megaprimer was then used in the second round of PCR using primer D5431 and pSR/*ogt102* as a template. Similarly, for *ogt1041*, primers ogt101 and pSRDown were used to amplify a megaprimer from pSR/*ogt104* template and the purified megaprimer was then used in the second round of PCR, with primer D5431 and pSR/*ogt104 as* template. Megaprimer PCR [18] was also employed to introduce the p35T, p34T and p11C substations into the *pogt1052* promoter fragment. This time, primers ogt1052 p35T, ogt1052 p34T and ogt1052 p11C were used with the downstream primer pSRDown and pSR/*ogt1052* as template. Purified megaprimers were then used in the second round of PCR with primer D5431 and pSR/*ogt1052*. All PCR products were restricted with EcoRI and HindIII, cloned into pSR and pRW50, and verified by Sanger sequencing.

The *ogt* promoter fragments used to investigate the effect of moving the location of NarL I on *ogt* promoter activity were all constructed from the *ogt1041* promoter fragment. For the *ogt104187* and *ogt104182* promoters, inverse PCR was used to introduce 10 and 5 bp of DNA downstream the *ogt1041* promoter NarL I site, using either forward primer ogt41 + 5 or ogt41 + 10 with reverse primer NarL inst DN and pSR/*ogt1041* as template. As PCR products carry a BglII site at each end, the product was restricted with BglII and self-ligated to produce plasmids pSR/*ogt104187* and pSR/*ogt104182*. For *ogt* derivatives ogt104172, ogt104167, ogt104162, ogt104157, ogt104152 and ogt104144 overlapping PCR was used. For each promoter, two DNA fragments were generated using an upstream primer (e.g. ogt4167 up) and primer D10527, and a downstream primer (e.g. ogt4167 DN) and primer D10520, with pRW50/*ogt1041* as template (see Supplementary Table S2). Purified PCR products were mixed together and extended by PCR to generate the full-length promoter. All *ogt1041* promoter derivatives were cloned into pRW50, using EcoRI and HindIII restriction sites.

### Construction of pDLC5

The BamHI-EcoRI fragment encoding the *narXL* operon and its regulatory region (226 bp upstream of the translation start site) was amplified from *E. coli* K-12 chromosomal DNA using the primers NarXPromoterFWD and NarLCTDReco. The fragment was cloned in to pLG339 to produce pDLC5. Megaprimer PCR [18] was used to introduce a single alanine substitution to amino acid residues 178 and 179 of the DNA fragment encoding NarXL to produce NarL-R178A and NarL-R179A. Plasmid pDLC5 was used as a template to amplify *narXL* using NarLCTDReco as a reverse primer with either NarL178AST or NarL179AST, respectively. The megaprimer from the first round PCR was used in the second round PCR with primer pLGFbamH. The purified product was cut with BamHI and EcoRI and cloned into pLG339.

### β-galactosidase assays

Plasmids containing *ogt::lacZ* promoter fusions were transformed into relevant strains and β-galactosidase activities were measured using the Miller protocol [19]. Single colonies containing the promoter::*lacZ* fusion were inoculated into Lennox Broth (2% (w/v) peptone (Oxoid), 1% (w/v) yeast extract (Oxoid) and 170 mM NaCl) and grown at 37°C overnight. To assay activities, overnight culture was inoculated into 10 ml of minimal salts media (as detailed in [15]) and grown at 37°C until the optical density (OD_650_) reached between 0.5 and 0.6. The media was supplemented with 20 mM sodium nitrate where appropriate. β-galactosidase activities are expressed as nmol ONPG (o-nitrophenyl-β-d-galactopyranose) hydrolysed min^−1^ mg^−1^ dry cell mass and represent the average of three independent experiments. Complete datasets are available at https://etheses.bham.ac.uk//id/eprint/4480/1/Ruanto13PhD.pdf

### NarL protein preparation and footprinting analysis

Preparation and purification of a fusion of maltose-binding protein to NarL (MBP-NarL) was as described by Li *et al*. [20]. In all experiments, the mature native NarL protein was used after the MBP moiety had been cleaved from MBP-NarL using protease factor Xa (New England Biolabs). To phosphorylate NarL, the protein was pre-incubated with 50 mM acetyl phosphate at 37°C for 45 min prior to use [21]. For footprinting experiments, EcoRI-HindIII promoter fragments were cloned into pGEM-Teasy (Promega) and purified plasmid was linearized by HindIII, treated with calf intestinal alkaline phosphatase (New England Biolabs) and then restricted with AatII. DNAse I footprinting was performed on P^32^ end-labelled AatI-HindIII fragments as in our previous work [21]. To monitor the location of αCTD in transcriptionally competent complexes at the *ogt* promoter, RNAP holoenzyme was reconstituted with α subunits that had been labelled with iron [S]-1-[p-bromoacetamidobenzyl]-ethylenediamine tetraacetic acid (FeBABE) at position 302, following the procedure by Lee *et al.* [22]. 200 nM of FeBABE-tagged RNAP was incubated at 37°C for 20 min with radiolabelled AatI-HindIII *ogt* promoter fragment and 3.2 µM phospho-NarL in 25 µl final volume of HEPES-glutamate buffer (20 mM HEPES (4-(2-Hydroxyethyl)piperazine-1-ethanesulfonic acid), 5 mM MgCl_2_, 50 mM potassium glutamate, 1 mM dithiothreitol) containing 0.5 mg/ml BSA. Sodium ascorbate and hydrogen peroxide was added to a final concentration of 5 mM and 0.06%, respectively, to start DNA cleavage. DNA cleavage patterns were analysed by electrophoresis on denaturing 6% acrylamide sequencing gels, containing 1×TBE (Tris-borate-EDTA), and were imaged using a Bio-Rad Molecular Imager FX and Quantity One software (Bio-Rad).

## Results

### NarL-dependent activation of the *ogt* promoter

In our previous study [15], we described the 326 bp *ogt100* DNA fragment that could be cloned into the low copy number pRW50 *lac* expression plasmid [17] to generate a fusion of the *E. coli* K-12 *ogt* promoter to the *lac* operon. Using this *ogt::lac* fusion, we showed that expression from the *ogt* promoter was induced by nitrate and this was dependent on NarL. Bandshift assays and footprinting experiments identified two DNA sites for NarL (NarL I and NarL II) and the proposed organisation of the *ogt* promoter is shown in Figure 1A. Our previous study [15] identified 7-2-7 sequences at both NarL I and NarL II, and, using the point mutations indicated in Figure 1A, we showed that both DNA sites for NarL were essential for nitrate-dependent NarL-mediated induction. Hence, induction was lost both with the *ogt102* fragment (derived from *ogt100*) that carried point mutations in NarL I, and with the *ogt104* fragment that carried point mutations in NarL II (Figure 1A–D) [15].

Since the NarL-activated *E. coli yeaR-yoaG* promoter contains a single 7-2-7 sequence, we reasoned that it might be possible to make an *ogt* promoter derivative that could be similarly activated. Hence, starting with the *ogt102* fragment (that lacks NarL I), we introduced several point mutations that made the NarL II site closer to the NarL consensus (i.e. *ogt1052*, illustrated in Figure 1B,C) [23]. Similarly, starting with the *ogt104* fragment (that lacks NarL II) we introduced several point mutations that made the NarL I site closer to the NarL consensus (i.e. *ogt1041*, illustrated in Figure 1B,C) [23]. Measurements of expression of the *ogt::lac* fusions with the new fragments cloned into pRW50 showed that *ogt* promoter activity was increased by the changes (Figure 1D). Hence, with the *ogt1052* fragment, which carried an improved 7-2-7 sequence at NarL II, promoter activity was substantially increased both in the presence and absence of nitrate, whilst with the *ogt1041* fragment, which carried an improved 7-2-7 sequence at NarL I, promoter activity was increased and was induced by nitrate.

**Figure 2.**
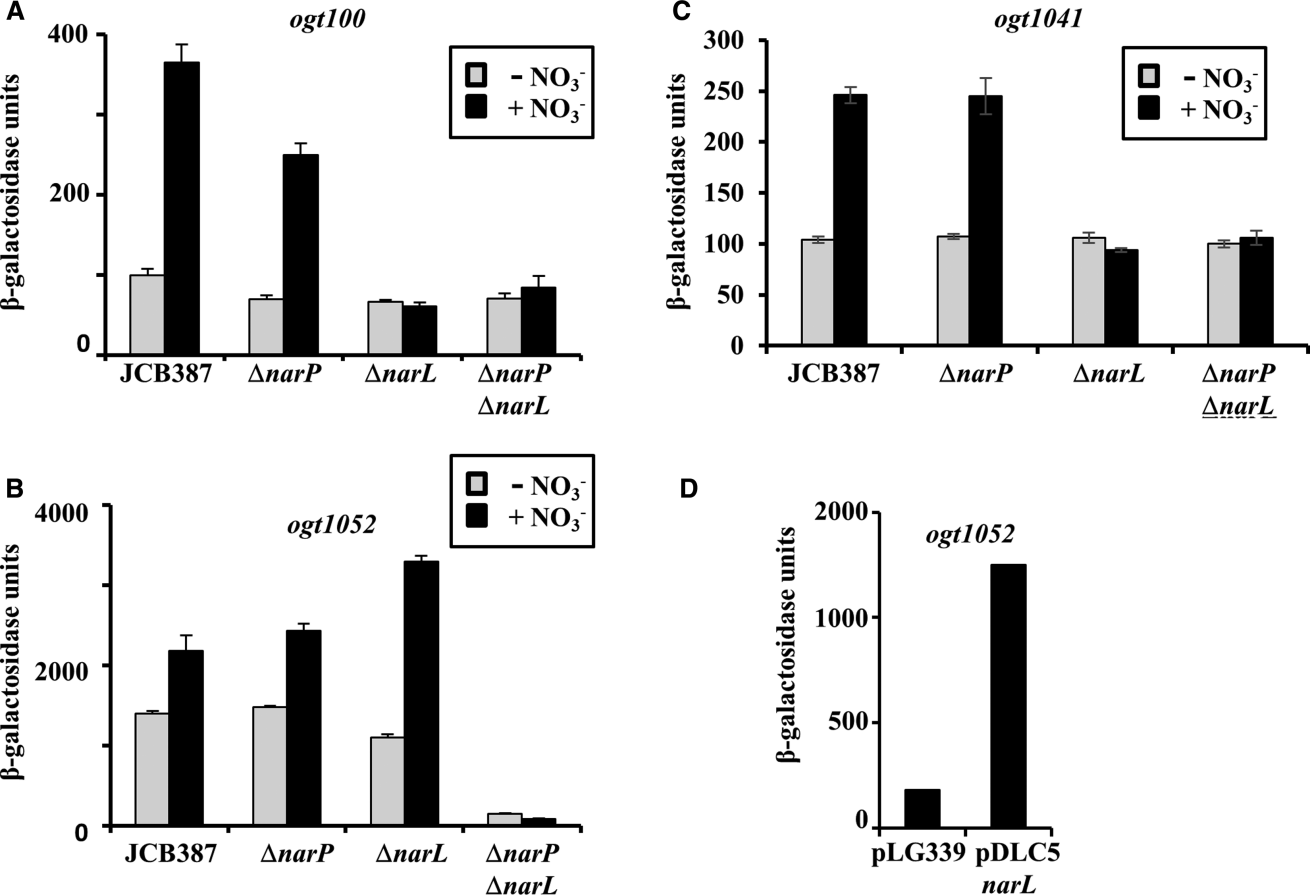
NarL- and NarP-dependent activation of *ogt* promoter fragments. The figure shows β-galactosidase activities measured in wild type JCB387, JCB3875 (Δ*narP*), JCB3883 (Δ*narL*) and JCB3884 (Δ*narL* Δ*narP*) cells, carrying the (**A**) *ogt100*, (**B**) *ogt1052* and (**C**) *ogt1041* promoters, cloned into the *lacZ* expression vector pRW50. (**D**) The panel details β-galactosidase activities measured in JCB3884 (Δ*narL* Δ*narP*) cells carrying the *ogt1052* promoter, cloned into pRW50, with either pLG339 (empty vector control) or pDLC5 (*narXL*). For all panels, cells were grown in minimal salts media supplemented with 20 mM nitrate, where indicated (black-filled bars). β-galactosidase activities are expressed as nmol ONPG hydrolysed min^−1^ mg^−1^ dry cell mass and represent the average of three independent experiments.

To check the dependence on NarL of the promoters carried by the *ogt1052* and *ogt1041* fragments, we repeated the assays in a Δ*narL* host background. Because *E. coli* K-12 carries a NarL-paralogue, NarP, we also checked in a Δ*narP* background and a double Δ*narP* Δ*narL* background. Recall that NarP can substitute for NarL at a limited number of promoters that are regulated by NarL [7–9,14]. Data presented in Figure 2A–C show that, with the starting *ogt100* promoter, nitrate-dependent induction of promoter activity was lost in the Δ*narL* and Δ*narP* Δ*narL* backgrounds, NarP has little or no effect, and a similar pattern is seen with the *ogt1041* promoter that carried a single improved 7-2-7 sequence at NarL I. In contrast, with the *ogt1052* promoter that carried a single improved 7-2-7 sequence at NarL II, whilst both nitrate-dependent and nitrate-independent activity was reduced to background levels in the double Δ*narP* Δ*narL* background, it was but subtly affected when only *narL* or *narP* was disrupted (Figure 2B). As a control, we used the pDLC5 plasmid, that carries a functional *narL* gene to restore induction in the Δ*narP* Δ*narL* background (Figure 2D).

The data in Figure 2 show that expression from the starting *ogt* promoter and the derivative (*ogt1041*) carrying a single improved NarL I site, was activated by nitrate in a NarL-dependent manner and that NarP is unable to substitute for NarL. In contrast, the *ogt1052* promoter derivative, carrying a single improved NarL II site, appears to be activated by either NarL or NarP, and substantial activity can be measured in the absence of added nitrate to the bacterial growth medium (Figure 2B). We suppose that, even in the absence of added nitrate, there is a basal level of NarL/NarP phosphorylation, and this must be sufficient to explain the measured activity, but we were concerned that the relatively high expression levels (compared with *ogt100* or *ogt1041*) might be due to the accidental creation of a new promoter. To investigate this, we exploited the observation that a consensus A base is essential as the second base on the non-template strand of the −10 hexamer at *E. coli* promoters that are served by RNAP carrying the housekeeping σ factor [24]. Hence, the −10 hexamer of the *ogt1052* promoter was changed from 5′-TATCTT-3′ to 5′-TCTCTT-3′. Results illustrated in Supplementary Figure S1 showed that this change (denoted p11C) completely suppresses promoter activity, strongly arguing that the measured promoter activity of the *ogt1052* fragment is due to a single promoter.

### A nitrate-dependent promoter with a novel architecture

**Figure 3.**
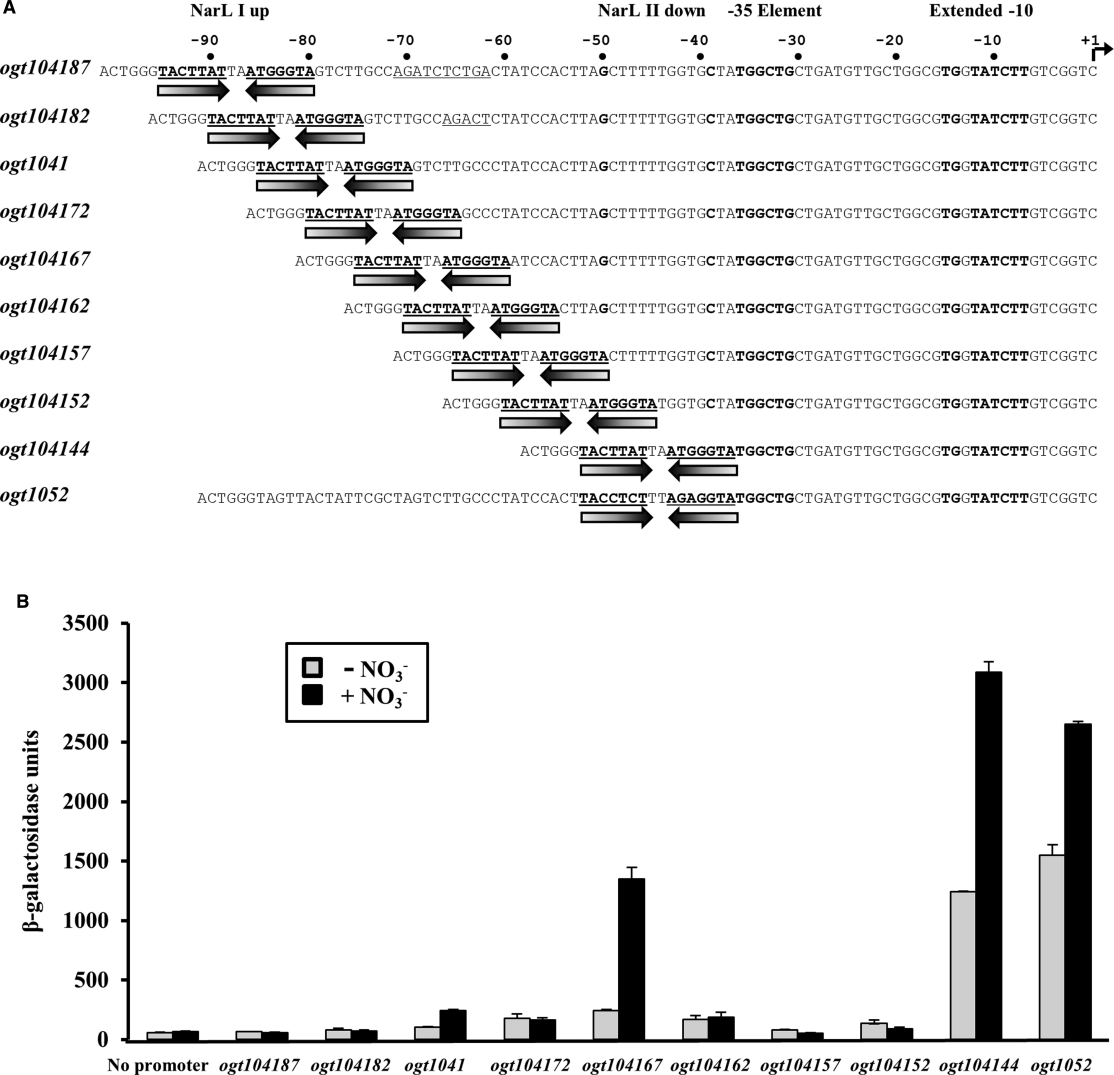
Position-dependent effects of altering the location of NarL I at the *ogt1041* promoter. (**A**) The panel shows the partial base sequences of promoters, based on the *ogt1041* promoter, in which the improved NarL I binding site was moved to different positions. Inverted arrows show the location of NarL I in each fragment and the relevant sequence is bold and underlined. The sequence is numbered with respect to the transcript start site (+1), which is indicated by a bent arrow. The extended −10 and −35 elements are also indicated and in bold. (**B**) The panel shows β-galactosidase activities measured in wild type *E. coli* K-12 JCB387 cells, carrying the promoters detailed in (**A**) as *lacZ* fusions, cloned into expression vector pRW50 [17]. Cells were grown in minimal salts media supplemented with 20 mM sodium nitrate, where indicated. β-galactosidase activities are expressed as nmol ONPG hydrolysed min^−1^ mg^−1^ dry cell mass and represent the average of three independent experiments.

One of our objectives was to develop a simple NarL-regulated promoter that could be exploited for synthetic biology applications. The promoter carried by the *ogt1041* fragment, with a single 7-2-7 DNA site for NarL centred at position −77.5 (i.e. between base pairs 77 and 78 upstream of the transcript start) appeared to be too weak for this purpose. Likewise, the *ogt1052* promoter (with a single 7-2-7 DNA site for NarL centred at position −44.5) was unsuitable, as the expression was not coupled to nitrate. However, our observation that NarL could activate transcription from 7-2-7 sequences at two different positions (−77.5 and −44.5) encouraged us to construct a family of promoter fragments, related to the *ogt1041* and *ogt1052* promoters, with the single 7-2-7 DNA site at different locations (Figure 3A) and the promoter activity of each fragment was assayed as above. Whilst most of the constructions resulted in poorly active promoters, the *ogt104167* fragment, which carries a single 7-2-7 DNA site for NarL centred at position −67.5, displays clear nitrate-inducible promoter activity (Figure 3B) and this activity is dependent on NarL and on the 5′-TATCTT-3′ −10 hexamer element (data not shown). The coupling of transcription to the addition of nitrate seen with this promoter suggests that it might be useful for future exploitation [25–27].

### Interactions between NarL and the RNA polymerase α subunit C-terminal domain during activation at the *ogt* promoter

The N- and C-terminal domains of each of the RNAP α subunits is joined by a flexible linker, and this accounts for the variety of architectures seen at many bacterial promoters subject to regulation by transcription factors that function by making contact with αCTD [1,3]. The observation that NarL could activate transcription when a 7-2-7 DNA target site for NarL was positioned at three different locations upstream from the *ogt* core promoter region prompted us to investigate whether the αCTD of RNAP could be a target for NarL. To do this, we measured the effect of introducing plasmids encoding the RNAP α subunit, with single alanine substitutions in αCTD, upon NarL-dependent activation of the *ogt100, ogt1052* and *ogt1041* promoters. We used a set of 67 plasmids each encoding α with a unique alanine substitution in αCTD between positions 255 and 329 of α, as in our previous work [28–30]. The experiment works on the principle that sufficient RNAP molecules carrying alanine substitutions will assemble, such that, if a particular residue in αCTD is important for transcription activation by NarL, NarL function will be compromised. Each plasmid encoding α with a particular alanine substitution was introduced into Δ*narP* host cells carrying the *ogt100::lac*, *ogt1052::lac* or *ogt1041::lac* fusions borne on pRW50.

**Figure 4.**
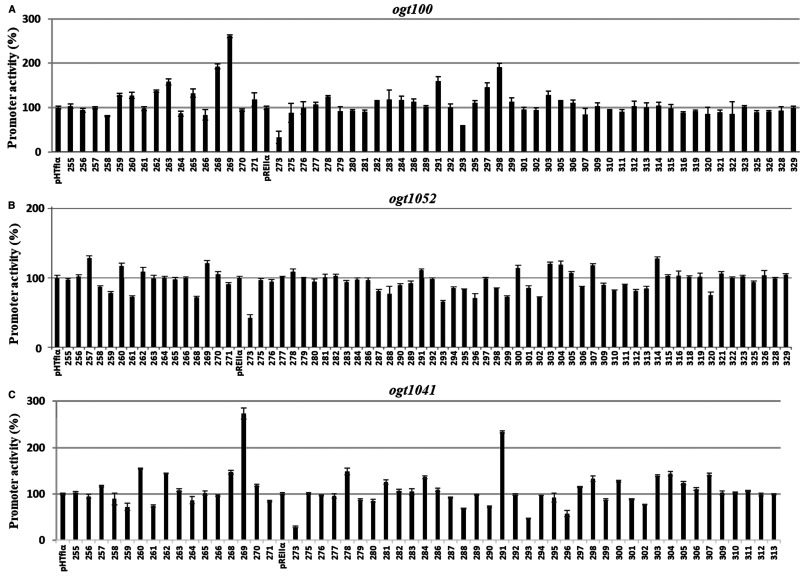
Residues of αCTD required for transcription activation at *ogt* promoter derivatives. The figure shows β-galactosidase activities measured in JCB3875 (Δ*narP*) cells that carried either (**A**) *ogt100*, (**B**) *ogt1052* or (**C**) *ogt1041* cloned into pRW50, together with pHTf1 or pREII plasmids, encoding derivatives of α with a single alanine substitution (residues 255−329). Cells were grown in minimal salts media supplemented with 20 mM sodium nitrate and promoter activities are presented as percentages of the activity measured in cells that carry plasmids encoding the wild type α subunit.

**Figure 5.**
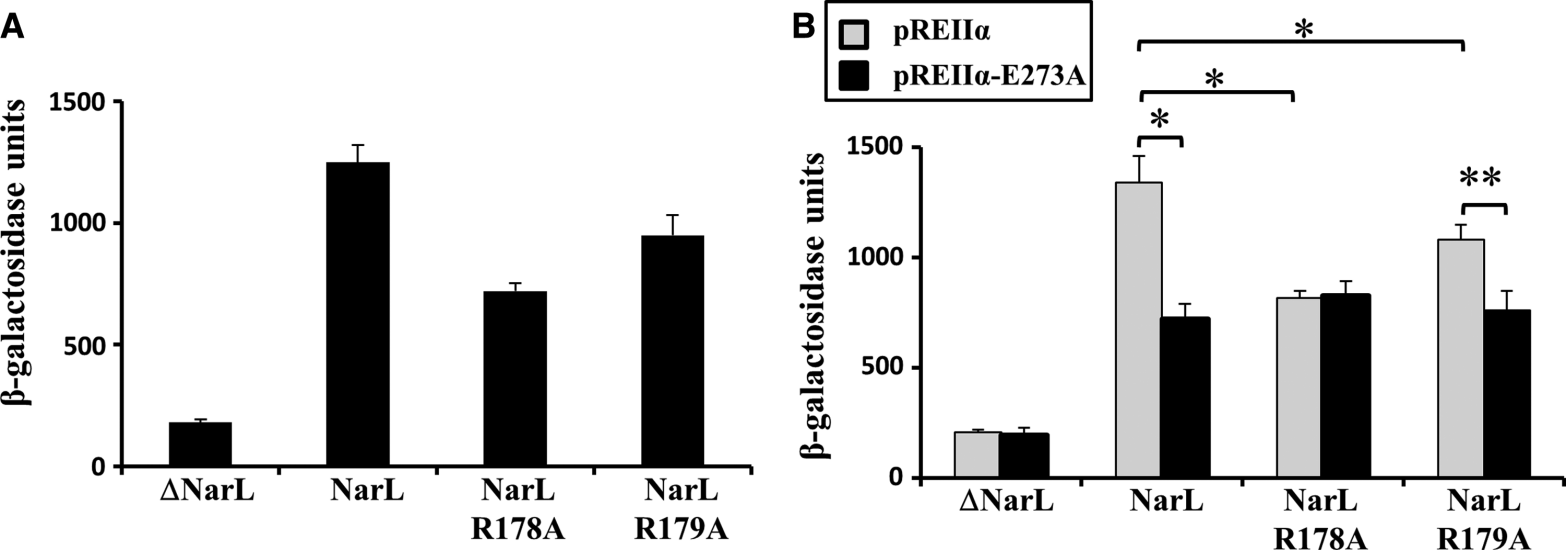
Epistasis studies of the interaction between NarL and the αCTD of RNAP at the *ogt1052* promoter. Panels (**A**) and (**B**) show β-galactosidase activities measured in JCB3884 (Δ*narL* Δ*narP*) cells, which contain pRW50/*ogt1052* and pDLC5, carrying various NarL derivatives. In (**B**) cells also contain either pREIIα (encoding the wild type α subunit) or pREIIα-273 (encoding an α derivative with an alanine substitution at residue 273). In both cases, cells were grown in minimal salts media supplemented with 20 mM nitrate. β-galactosidase activities are expressed as nmol ONPG hydrolysed min^−1^ mg^−1^ dry cell mass and represent the average of three independent experiments. In panel (**B**), * indicates *P *< 0.05 and ** *P* = 0.05 (Student's *t-*test).

Data illustrated in Figure 4, show the effect of each alanine substitution on the activity of each *ogt* promoter derivative. Changes are seen with many substitutions, but the largest effect, seen at all three promoters, was caused by alanine substitution of α residue 273, which carries a surface-exposed sidechain, E273. A previous study [31] had argued that residues R178 and R179 of NarL form an activating region that interacts with RNAP to activate transcript initiation at target promoters. To investigate possible interactions between NarL R178 or R179 and RNAP α subunit residue E273, we introduced plasmid pDLC5, encoding wild type NarL or NarL with alanine substitutions at either position 178 or 179, into a Δ*narL* Δ*narP* host strain carrying the *ogt1052::lac* fusion in pRW50. Results illustrated in Figure 5A confirm that substitution of R178 or R179 in NarL compromises the ability of NarL to activate transcription. We then measured the effect of introducing a third plasmid, encoding the RNAP α subunit carrying alanine at position 273, on *ogt1052* promoter activity. Results illustrated in Figure 5B show that the α273 alanine substitution reduced activity with wild type NarL or NarL 179A, but caused no reduction with NarL 178A. This shows that the E273A substitution in the RNAP α subunit is epistatic to the R178A substitution in NarL, suggesting that, during NarL-dependent transcription activation, residue R178 of NarL may directly interact with α residue E273 in αCTD, at least at the *ogt1052* promoter.

**Figure 6.**
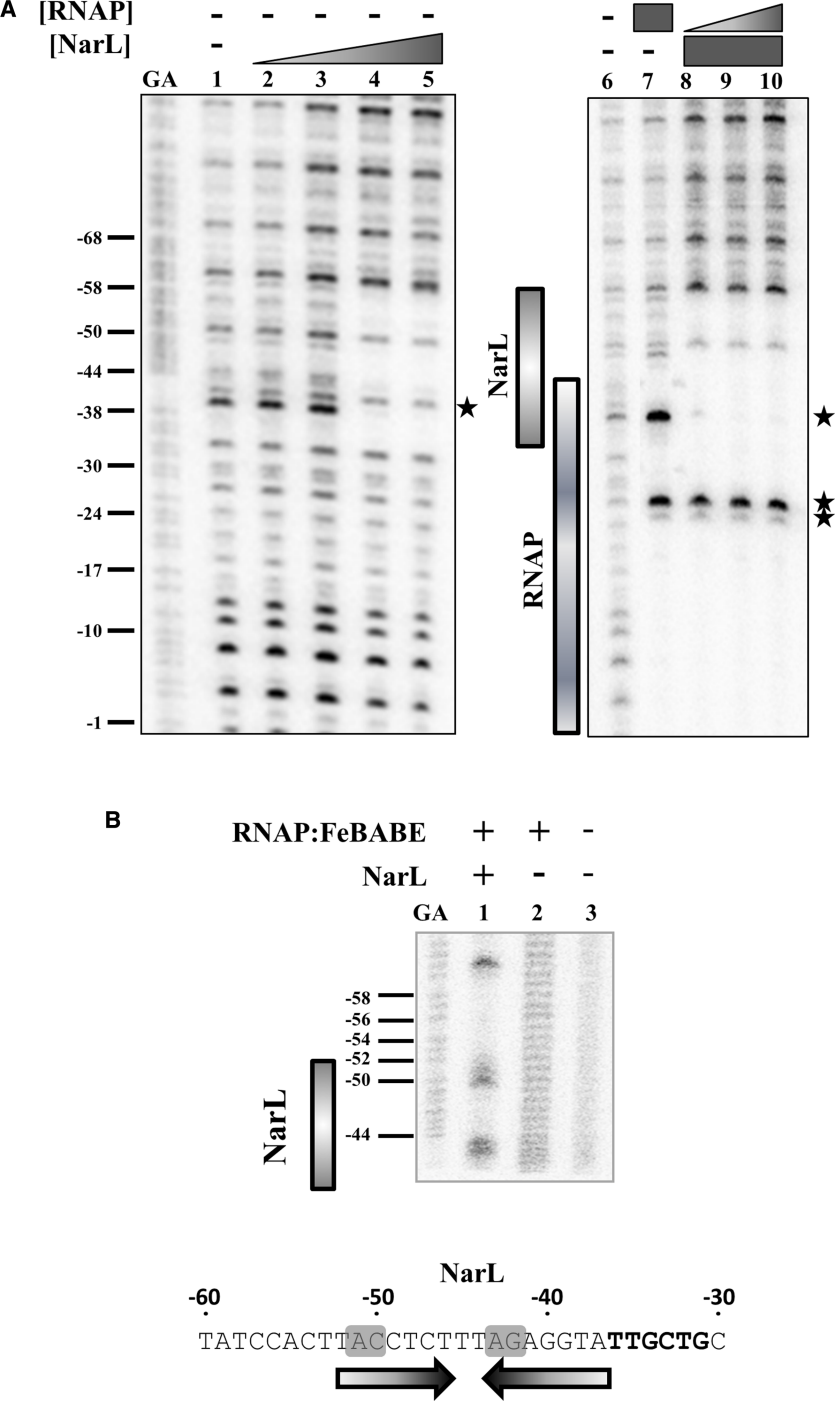
*In vitro* analysis of NarL and RNAP binding to the *ogt1052* p35T promoter. (**A**) The binding of NarL and RNAP to the *ogt1052* p35T promoter was investigated using DNAse I footprinting. Different concentrations of phospho-NarL and RNAP were incubated with P^32^ end-labelled *ogt1052* p35T promoter fragment and treated with DNAse I. The concentrations of NarL was as follows: lanes 1, 6 and 7, no protein; lane 2, 0.4 µM; lane 3, 0.8 µM; lanes 4 and 8 to 10, 1.6 µM; lane 5, 3.2 µM. The concentrations of RNAP holoenzyme was: lanes 1 to 6, no protein; lane 7 and 10, 160 nM; lane 8, 40 nM; lane 9, 100 nM. The extent of NarL and RNAP protections is indicated by grey boxes and hypersensitivity sites are starred. (**B**) The panel shows DNA cleavage patterns resulting from an FeBABE experiment with the P^32^ end-labelled *ogt1052* p35T promoter fragment and RNAP carrying an FeBABE attached to residue 302 of the α subunits of RNA polymerase. The reactions contained the following proteins: Lane 1, RNAP FeBABE and 3.2 µM phosho-NarL; Lane 2, RNAP FeBABE; Lane 3, no protein. The DNA sequence of the NarL site in the *ogt1052* p35T fragment is shown and the bases modified by FeBABE footprinting are shaded grey. In both panels, gels were calibrated using Maxam–Gilbert G + A sequence reactions (GA) and relevant positions are indicated.

To investigate the arrangement of αCTD and NarL during transcription activation by NarL, we used DNAse I footprinting with purified *E. coli* RNAP and NarL. Our initial attempts with the *ogt1052* promoter proved unsuccessful, as RNAP did not associate with the promoter *in vitro.* To overcome this, we exploited a derivative of the *ogt1052* promoter carrying the p35T mutation that changes the promoter −35 element from 5′-TGGCTG-3′ to 5′-TTGCGG-3′ [32], which better resembles the consensus, 5′-TTGACA-3′ (Supplementary Figure S2). The *ogt1052* promoter carrying the p35T mutation is still activated by NarL, but has a higher basal level of NarL-independent activity (Supplementary Figure S2). Thus, a purified P^32^ end-labelled DNA fragment carrying the *ogt1052* p35T promoter was incubated with increasing concentrations of purified phosphorylated NarL and subjected to partial DNAse I digestion. The results, illustrated in Figure 6A, show that NarL binding resulted in a clear footprint, due to binding at the 7-2-7 target site, centred at position −44.5 (lanes 2–5). Binding of RNAP alone also produced a footprint up to position −44 (lane 7), whilst co-incubation of NarL with RNAP resulted in an extended footprint (lanes 8–10). The pattern of protected and non-protected bands suggest that NarL and RNAP co-occupy the promoter DNA, but no RNAP-dependent protection is observed upstream of the protection due to NarL.

To locate the position of αCTD in the ternary NarL-RNAP-*ogt1052* p35T promoter complex, we exploited the inorganic DNA cleavage reagent FeBABE. Previously, we described the reconstitution of *E. coli* RNAP using α subunits that had been engineered to carry a single Cysteine at position 302, which was subsequently labelled with FeBABE [22]. Hence, the footprinting experiment was repeated using this preparation of RNAP, and, rather than using DNAse I, we used ascorbic acid and hydrogen peroxide to generate a pulse of hydroxyl radicals emanating from the bound FeBABE. This results in a footprint of local DNA cleavage that reflects the position of αCTD in the ternary NarL–RNAP–promoter complex. Data illustrated in Figure 6B show the appearance of bands due to cleavage near positions −43 and −51. This pattern of cleavage is similar to what we have previously observed at factor-independent promoters where αCTD binds to the DNA immediately upstream of σ Domain 4 that interacts with the promoter −35 element [22,33,34]. Note that the appearance of these bands, and hence the positioning of αCTD is dependent on NarL.

**Figure 7.**
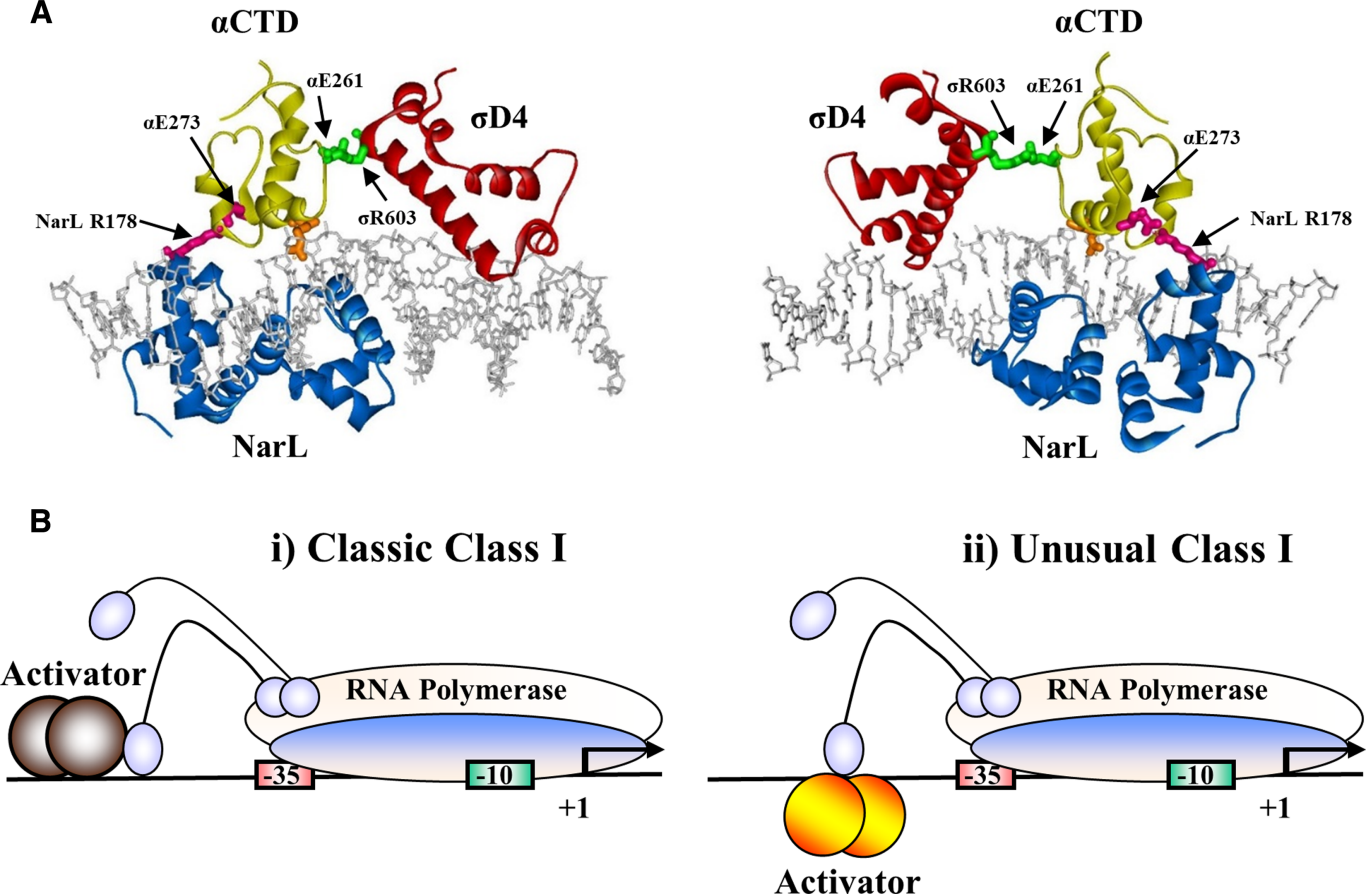
Structure-based model of the transcript initiation complex at the *ogt p*romoter. (**A**) The panel shows two views of a structure-based model detailing the binding of a NarL dimer, an αCTD of RNAP and Domain 4 of the σ subunit of RNAP (σD4) bound to NarL II at the *ogt1052* promoter. The αCTD is located immediately upstream of the boundary of the −35 hexamer, thus, it orients residue E273 to interact with residue R178 of NarL, which is located at position −45.5 on the opposite face of DNA helix from αCTD. This action causes residue 261 of αCTD to point downstream toward the σ subunit and interact with R603 in Domain 4 of the σ subunit [41,42]. The model was created using WebLab Viewer (Accelrys). (**B**) The panel shows the architectures of (**i**) simple and (**ii**) unusual Class I activator-dependent promoters. At ‘classic’ Class I promoters, a single transcription factor, bound to an upstream site, interacts with the αCTD of RNAP on the same face of the DNA helix to recruit RNAP to the promoter. At unusual Class I promoters (e.g. the *ogt1052* promoter), transcription factors, such as NarL, bind to the opposite face of the DNA helix to contact αCTD and recruit polymerase.

Figure 7A illustrates a molecular model, showing the juxtaposition, suggested by our results, of Domain 4 of the RNAP σ subunit, the C-terminal domain of one RNAP α subunit, and two NarL DNA binding domains, bound at the *ogt1052* promoter. The model was assembled from published structures of NarL bound to a 7-2-7 element [31,35], and of σ Domain 4, bound to a promoter −35 element, contacting αCTD, positioned immediately adjacent, as found at both activator-dependent and activator-independent promoters [36,37]. With this arrangement, residue E273 of αCTD can make a direct interaction with R178 of the upstream bound NarL and we propose that this interaction plays a role in NarL-dependent recruitment of RNAP to the *ogt* promoter.

## Discussion

The *E. coli ogt* gene encodes a DNA repair enzyme, O^6^-alkylguanine-DNA-alkyltransferase, whose role is to remove alkyl groups from guanine bases in chemically damaged DNA [38]. The accumulation of reactive nitrogen species during the metabolism of nitrate can trigger alkylation of DNA, and we have argued that the dependence of the *ogt* promoter on nitrate ions (via NarL) suggests that *ogt* expression is part of a prophylactic response that limits DNA damage when nitrate is being used as a growth substrate [15]. NarL appears to activate the *ogt* promoter by interacting directly with RNAP, thereby recruiting RNAP to the promoter. Recruitment at the ‘wild-type’ *ogt* promoter requires tandem-bound NarL dimers and we previously showed that this renders the promoter subject to repression by Fis protein via a DNA site for Fis that overlaps the upstream DNA site for NarL (NarL I). Levels of Fis are known to rise sharply during rapid bacterial growth [39] and we reasoned that nitrate-induced DNA damage was less critical, and could be better tolerated, in these conditions [15].

In this study, we have shown that changing the base sequence of either the upstream DNA site for NarL (NarL I centred at position −77.5), or the downstream site (NarL II centred at position −44.5), to the consensus 7-2-7 sequence [23], simplifies the *ogt* promoter so that it becomes dependent on the binding of just one NarL dimer. Furthermore, we showed that NarL was also able to activate transcription when located at position −67.5 (i.e. the *ogt104167* promoter). Comparison of *ogt* promoter sequences in different *E. coli* strains shows that the arrangement with tandem DNA sites for NarL is well conserved (Supplementary Figure S3). However, in some pathogenic *E. coli* strains, the downstream NarL II is mutated and there is ∼10 bp deletion between NarL I and NarL II, making these promoters more reminiscent of the *ogt104167* promoter (Supplementary Figure S4). Additionally, we found *ogt* promoters from many *Salmonella* species that resembled the *ogt1052* promoter, as at these promoters, the upstream NarL DNA site is missing and the downstream site appears to be improved (Supplementary Figure S5). Hence, the constructions that we report here as new are in fact not novel, but have arisen during the evolution of the *ogt* promoter in different Enterobacteriaceae. Interestingly, the location of the DNA site for NarL at our new promoters affected the performance of the promoter in terms of activity, dependence on nitrate, and the ability to be activated by NarP.

Transcription activation at many bacterial promoters involves the binding of an activator protein to an operator target, followed by direct activator–RNAP interactions that help recruit the RNAP to the promoter, so it is correctly placed to orchestrate transcript initiation [1,3]. Many such activators interact with the RNAP αCTD, with the activator binding upstream of αCTD on the same face of the promoter DNA (illustrated in Figure 7Bi). This is often called Class I activation, and many studies have shown that, because αCTD is connected to the RNAP α subunit N-terminal domain (αNTD) by a flexible linker, the activator can be positioned at different upstream locations [1,3]. The results presented here argue strongly that this is the case for activation of the *E. coli ogt* promoter by NarL bound as a dimer at sites centred at positions −77.5, −67.5 and −44.5. However, at least when at position −44.5, NarL is bound to the opposite face of the promoter DNA as αCTD and σ Domain 4 (Figure 7A). We suggest that this arrangement is a variation from the simple models for Class I activation [1] and reflects that activators that function by recruitment mechanisms can use any available contact with any available RNAP surface (Figure 7Bii). We note that a similar arrangement has been proposed at the bacteriophage lambda P_RE_ promoter, during activation by lambda cII protein, with cII binding on the opposite side of the DNA to σ Domain 4 and αCTD, and making direct contact with αCTD rather than σ Domain 4 [33,40]. Future studies with NarL will focus on investigating its ability to activate from upstream locations (Figure 3), understanding differences from NarP (residues R178 and R179 are not conserved), and exploiting it for biotechnology applications.

## Open Access

Open access for this article was enabled by the participation of University of Birmingham in an all-inclusive *Read & Publish* pilot with Portland Press and the Biochemical Society under a transformative agreement with JISC.

## Abbreviations

bp: base pairs
FeBABE: iron [S]-1-[p-bromoacetamidobenzyl]-ethylenediamine tetraacetic acid
HEPES: 4-(2-Hydroxyethyl)piperazine-1-ethanesulfonic acid
NAPs: nucleoid-associated proteins
ONPG: o-nitrophenyl-β-d-galactopyranose
RNAP: RNA polymerase
TBE: tris-borate-EDTA
αCTD: C-terminal domain of the RNAP α subunit
αNTD: N-terminal domain of the RNAP α subunit
