# Supplementary material for: Activation by NarL at the Escherichia coli ogt promoter
Source: Biochem J. 2020 Aug 7;477(15):2807–20. doi: 10.1042/BCJ20200408 (PMC7419079; doi:10.1042/BCJ20200408)
Supplement: Supplementary Tables S1-S2 and Figures S1-S5 [file BCJ-477-2807-s1.pdf]

Supplementary Data.

Supplementary Table S1. Strains, plasmid and promoters used in the study

Name	Details	Source
**Strains.**		
JCB387	Δ*nir*, Δ*lac*	[1]
JCB3875	JCB387 Δ*narP252*::*Tn1*0d(Cm)	[1]
JCB3883	JCB387 Δ*narL*	[2]
JCB3884	JCB387 Δ*narL* Δ*narP252*::*Tn1*0d(Cm)	[2]
**Plasmids.**		
pRW50	Broad-host-range *lacZ* expression vector for cloning EcoRI-HindIII fragments. Encodes tetracycline resistance.	[3]
pSR	pBR322 derivative cloning vector, containing a λ *oop* transcription terminator. Encodes ampicillin resistance.	[4]
pLG339	Cloning vector encoding kanamycin and tetracycline resistance.	[5]
pDLC5	Derivative of pLG339 containing BamHI-EcoRI fragment carrying *narXL* operon and regulatory region.	This work
pGEM-Teasy	Vector used for the cloning of PCR products and blue/ white screening of recombinants. Encodes ampicillin resistance.	Promega
pREIIα, pHTf1α and derivatives	Plasmid carrying *rpoA* encoding RNAP α subunit and derivatives, carrying single alanine substitutions at positions 255–329.	[6-8]
**Starting promoter fragments.**		
ogt100	*E. coli ogt* promoter fragment carrying sequence from -269 to +51.	[9]
ogt102	*E. coli ogt100* promoter fragment carrying C to G and T to C substitutions at position -83 and -72.	[9]
ogt104	*E. coli ogt100* promoter fragment carrying T to G and G to C substitutions at position -50 and -39.	[9]

Supplementary Table S2. Primers used in this study

Primer name	Sequence (5` to 3`)
D5431	ACCTGACGTCTAAGAAACC
pSRDown	ATCCAGATGGAGTTCTGAGG
D10520	CCCTGCGGTGCCCCTCAAG
D10527	GCAGGTCGTTGAACTGAGCCTGAAATTCAGG
ogt101	CCAGACGCAAACTGGGTACTTATTAATGGGTAGTCTTGCCC
ogt105	GCCCTATCCACTTACCTCTTTAGAGGTATGGCTGCTGATG
ogt1052 p35T	GTATTGCTGCTGATGTTGCTGGCGT
ogt1052 p34T	GTATGTCTGCTGATGTTGCTGGCGT
ogt1052 p11C	TTGCTGGCGTGGTCTCTTGTCGGTCTGCCG
ogt41 +5	GGG**AGATCT**ATCCACTTAGCTTTTTGGTGCTA
ogt41 +10	GGG**AGATCT**CTGATATCCACTTAGCTTTTTGGTGCTA
NarL I inst DN	CCC**AGATCT**GGGCAAGACTACCCATTAATA
ogt4172 up	ACTTATTAATGGGTAGCCCTATCCACTTAG
ogt4172 DN	CTAAGTGGATAGGGCTACCCATTAATAAGT
ogt4167 up	ACTTATTAATGGGTAATCCACTTAGCTTTT
ogt4167 DN	AAAAGCTAAGTCGATTACCCATTAATAAGT
ogt4162 up	ACTTATTAATGGGTACTTAGCTTTTTGGTG
ogt4162 DN	CACCAAAAAGCTAAGTACCCATTAATAAGT
ogt4157 up	ACTTATTAATGGGTACTTTTTGGTGCTATG
ogt4157 DN	CATAGCACCAAAAAGTACCCATTAATAAGT
ogt4152 up	ACTTATTAATGGGTATGGTGCTATGGCTGC
ogt4152 DN	GCAGCCATAGCACCATACCCATTAATAAGT
ogt4144 up	ACTTATTAATGGGTATGGCTGCTGATGTT
ogt4144 DN	CAACATCAGCAGCCATACCCATTAATAAGT
NarXPromoterFWD	GG**GGATCC**ACCCATAGTGAGTACAGTGACT
NarLCTDReco	CC**GAATTC**TCAGAAAATGCGCTCCTGATGCACCCA
NarL178AST	AAGATGATTGCCGCCCGCCTGGATATCACC
NarL179AST	ATGATTGCCCGCGCCCTGGATATCACCGAA
pLGFbamH	GAGCGGCGACGATAGTCATG

Supplementary Figure Legends.

Supplementary Figure S1. Disruption of the -10 and -35 promoter elements in the *ogt1052* promoter fragment. (**A**) The panel shows partial base sequence of the *ogt1052, ogt1052* p11C and *ogt1052* p34T promoter derivatives, from position -52 to +1. The -10 and -35 promoter elements are in bold and an inverted arrow indicates the improved NarL II site, carried by the *ogt1052* promoter fragment. The p11C and p34T substitutions, which disrupt the -10 and -35 promoter elements [10], respectively, are underlined. Measured β-galactosidase activities are also shown for (**B**) wild-type JCB387 and (**C**) JCB3884 (Δ*narL* Δ*narP*) cells, carrying the *ogt1052*, *ogt1052* p11C and *ogt1052* p34T promoter fragments cloned into the *lacZ* expression vector pRW50. Cells were grown in minimal salts media supplemented with 20 mM sodium nitrate, where indicated. β-galactosidase activities are expressed as nmol ONPG hydrolysed min^{-1} mg^{-1} dry cell mass and represent the average of three independent experiments.

Supplementary Figure S2. Improvement of the -35 promoter element in the *ogt1052* promoter fragment. (**A**) The panel shows partial base sequence of the *ogt1052 and ogt1052* p35T promoter derivatives, from position -52 to +1. The -10 and -35 promoter elements are in bold and an inverted arrow indicates the improved NarL II site, carried by the *ogt1052* promoter fragment. The p35T substitution, which improves the -35 promoter element with respect to the -35 element consensus [10], is underlined. Measured β-galactosidase activities are also shown for (**B**) wild-type JCB387 and (**C**) JCB3884 (Δ*narL* Δ*narP*) cells, carrying the *ogt1052* and *ogt1052* p35T promoter fragments cloned into the *lacZ* expression vector pRW50. Cells were grown in minimal salts media supplemented with 20 mM sodium nitrate, where indicated. β-galactosidase activities are expressed as nmol ONPG hydrolysed min^{-1} mg^{-1} dry cell mass and represent the average of three independent experiments.

Supplementary Figure S3. Alignment of *ogt* promoter sequences from different *E. coli* strains. The figure shows the sequence of the *E. coli* K-12 strain MG1655 *ogt* promoter, aligned with the corresponding *ogt* promoter regions from various *E. coli* strains. The location of the NarL I and NarL II binding sites, the extended -10 element and the *ogt* transcription (+1) and translation (ATG) start sites is indicated. The differences between the MG1655 *ogt* promoter sequences and other *E. coli* strains are highlighted in red. DNA sequences were obtained from xBASE (http://xbase.warwick.ac.uk/) [11] and EcoCyc (https://ecocyc.org/) [12]. The strains used were as follows: Enterotoxigenic *E. coli* (ETEC) strain H10407, Enteroaggregative *E. coli* (EAEC) *strain* 042, EAEC strain 55989, *E. coli* B

strain BL21(DE3), enterohaemorrhagic *E. coli* (EHEC) strain O157:H7 Sakai and *E. coli* K-12 strain MG1655.

Supplementary Figure S4. Alignment of the *E. coli* K-12 *ogt* promoter sequence with those from various pathogenic *E. coli* strains. The figure shows the sequence of the *E. coli* K-12 strain MG1655 *ogt* promoter, aligned with the corresponding *ogt* promoter regions from various pathogenic *E. coli* strains. The location of the NarL I and NarL II binding sites, the extended -10 element and the *ogt* transcription (+1) and translation (ATG) start sites is indicated. The differences between the MG1655 *ogt* promoter sequences and other *E. coli* strains are highlighted in red. The NarL I and NarL II sites are aligned with the NarL consensus sequence (Y=C/T, M=A/C, K=G/T, R=A/G) [13]. DNA sequences were obtained from xBASE (http://xbase.warwick.ac.uk/) [11] and EcoCyc (https://ecocyc.org/) [12]. The strains used were as follows: Avian pathogenic *E. coli* (APEC) strain O1, Enteropathogenic *E. coli* (EPEC) strain E2348/69, Uropathogenic *E. coli* (UPEC) strain CFT073 and *E. coli* K-12 strain MG1655.

Supplementary Figure S5. Alignment of the *E. coli* K-12 and *Salmonella enterica ogt* promoter sequences. The figure shows the sequence of the *E. coli* K-12 strain MG1655 *ogt* promoter, aligned with the corresponding *ogt* promoter regions from various *Salmonella enterica* strains. The location of the NarL I and NarL II binding sites, the extended -10 element, and the *ogt* transcription (+1) and translation (ATG) start sites is indicated. The differences in sequence, between the *Salmonella enterica* and MG1655 in *NarL* II site, which improve NarL II in comparison to the NarL consensus sequence [13] are highlighted in pink (Y=C/T, M=A/C, K=G/T, R=A/G). DNA sequences were obtained from xBASE (http://xbase.warwick.ac.uk/) [11] and EcoCyc (https://ecocyc.org/) [12]. The strains used were as follows: *Salmonella enterica* serovar Typhimurium strain LT2, *Salmonella enterica* serovar Choleraesuis strain SC-B67, *Salmonella enterica* serovar Typhi strain CT18, *Salmonella enterica* serovar Dublin strain CT02021853, *Salmonella enterica* serovar Paratyphi A strain ATCC9150 and *E. coli* K-12 strain MG1655.

Supplementary Data References.

1 Page, L., Griffiths, L. and Cole, J.A. (1990) Different physiological roles of two independent pathways for nitrite reduction to ammonia by enteric bacteria. *Arch Microbiol*. **154**, 349-354

2 Tyson, K.L., Cole, J.A. and Busby, S.J. (1994) Nitrite and nitrate regulation at the promoters of two *Escherichia coli* operons encoding nitrite reductase: identification of common target heptamers for both NarP- and NarL-dependent regulation. *Mol Microbiol*. **13**, 1045-1055

3 Lodge, J., Fear, J., Busby, S., Gunasekaran, P. and Kamini, N.R. (1992) Broad host range plasmids carrying the *Escherichia coli* lactose and galactose operons. *FEMS Microbiol Lett*. **74**, 271-276

4 Kolb, A., Kotlarz, D., Kusano, S. and Ishihama, A. (1995) Selectivity of the *Escherichia coli* RNA polymerase E sigma 38 for overlapping promoters and ability to support CRP activation. *Nucleic Acids Res*. **23**, 819-826

5 Stoker, N.G., Fairweather, N.F. and Spratt, B.G. (1982) Versatile low-copy-number plasmid vectors for cloning in *Escherichia coli*. *Gene*. **18**, 335-341

6 Blatter, E.E., Ross, W., Tang, H., Gourse, R.L. and Ebright, R.H. (1994) Domain organization of RNA polymerase alpha subunit: C-terminal 85 amino acids constitute a domain capable of dimerization and DNA binding. *Cell*. **78**, 889-896

7 Tang, H., Severinov, K., Goldfarb, A., Fenyo, D., Chait, B. and Ebright, R.H. (1994) Location, structure, and function of the target of a transcriptional activator protein. *Genes Dev*. **8**, 3058-3067

8 Gaal, T., Ross, W., Blatter, E.E., Tang, H., Jia, X., Krishnan, V.V., Assa-Munt, N., Ebright, R.H. and Gourse, R.L. (1996) DNA-binding determinants of the alpha subunit of RNA polymerase: novel DNA-binding domain architecture. *Genes Dev*. **10**, 16-26

9 Squire, D.J., Xu, M., Cole, J.A., Busby, S.J. and Browning, D.F. (2009) Competition between NarL-dependent activation and Fis-dependent repression controls expression from the *Escherichia coli yeaR* and *ogt* promoters. *Biochem J*. **420**, 249-257

10 Browning, D.F. and Busby, S.J. (2016) Local and global regulation of transcription initiation in bacteria. *Nat Rev Microbiol*. **14**, 638-650

11 Chaudhuri, R.R., Loman, N.J., Snyder, L.A., Bailey, C.M., Stekel, D.J. and Pallen, M.J. (2008) xBASE2: a comprehensive resource for comparative bacterial genomics. *Nucleic Acids Res*. **36**, D543-546

12 Keseler, I.M., Mackie, A., Peralta-Gil, M., Santos-Zavaleta, A., Gama-Castro, S., Bonavides-Martinez, C., Fulcher, C., Huerta, A.M., Kothari, A., Krummenacker, M., Latendresse, M., Muniz-Rascado, L., Ong, Q., Paley, S., Schroder, I., Shearer, A.G., Subhraveti, P., Travers, M., Weerasinghe, D., Weiss, V., Collado-Vides, J., Gunsalus, R.P., Paulsen, I. and Karp, P.D. (2013) EcoCyc: fusing model organism databases with systems biology. *Nucleic Acids Res*. **41**, D605-612

13 Darwin, A.J., Tyson, K.L., Busby, S.J. and Stewart, V. (1997) Differential regulation by the homologous response regulators NarL and NarP of *Escherichia coli* K-12 depends on DNA binding site arrangement. *Mol Microbiol*. **25**, 583-595

Supplementary Figure S1.

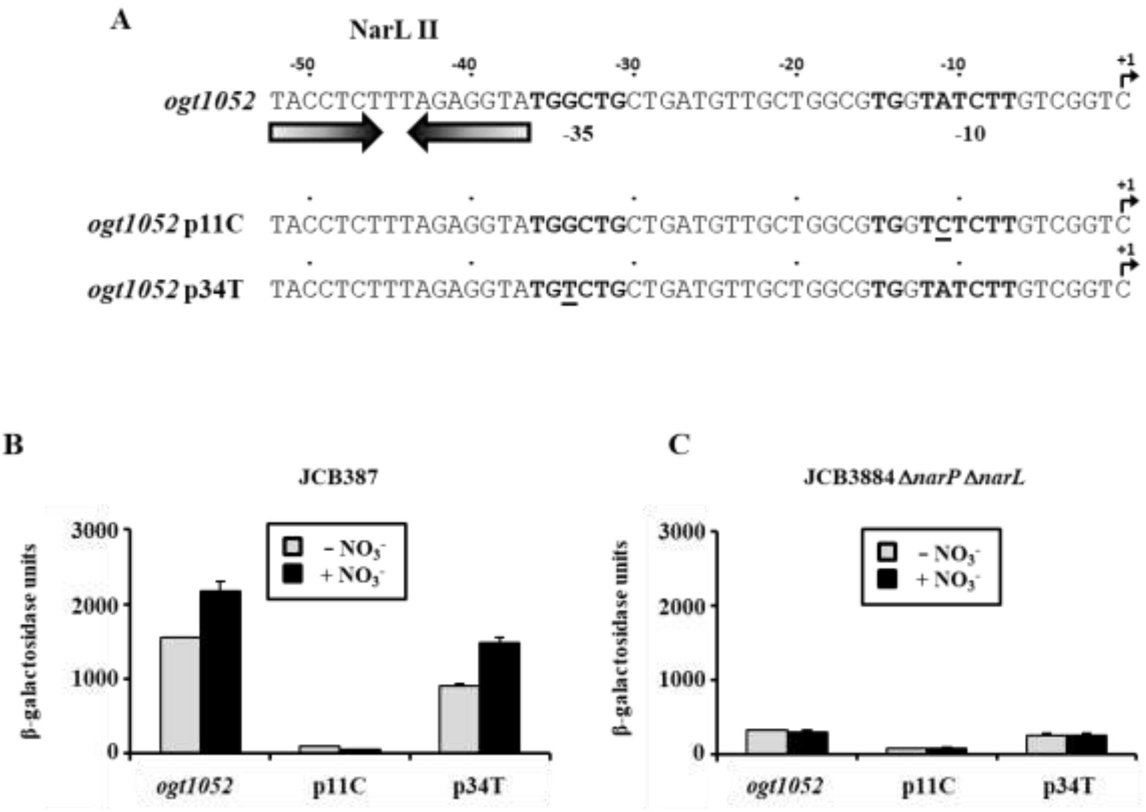

Supplementary Figure S2.

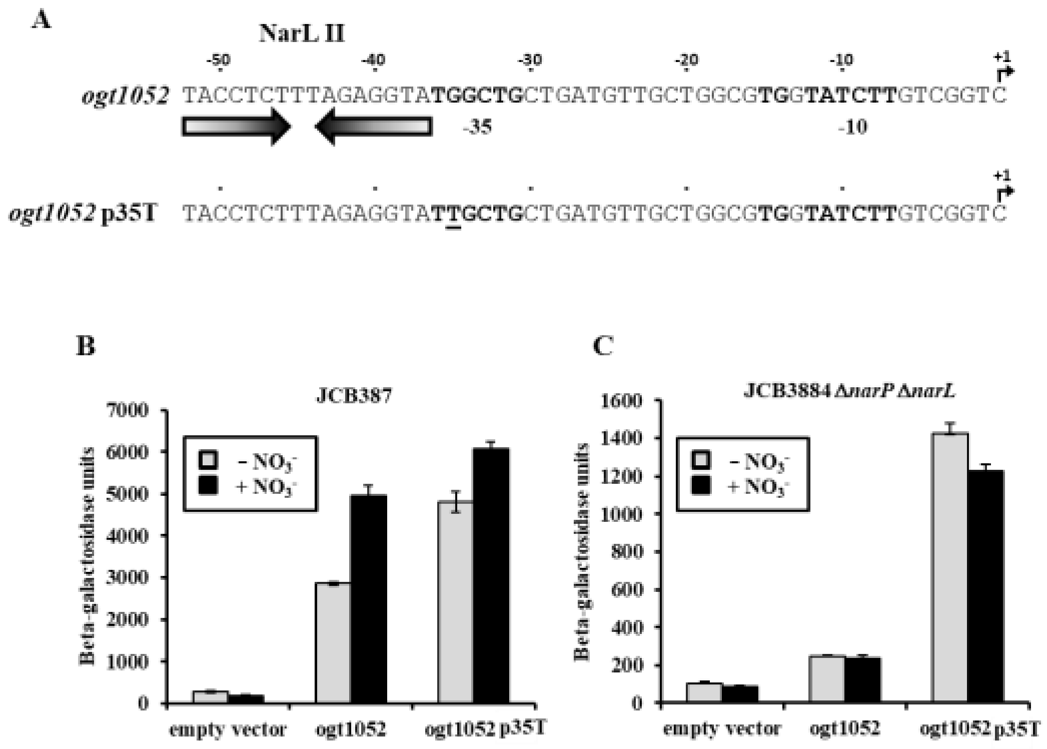

Supplementary Figure S3.

```
ogt ETEC H10407     ACGCTACAAACCAGACGCGAAACTGGGTACTTACTATTCGTTAGTCTTGCCCTATCCACT
ogt EAEC 042        ACGTTACAAACCAGACGCGAAACTGGGTACTTACTATTCGTTAGTTTTGCCCTATCCACT
ogt EAEC 55989      ACGCTACAAACCAGACGCGAAACTGGGTACTTACTATTCGTTAGTCTTGCCCTATCCGCT
ogt BL21(DE3)       ACGCTACAAACCAGACGCGAAACTGGGTACTTACTATTCGTTAGTCTTGCCCTATCCGCT
ogt EHEC Sakai      ACGCTACAAACCAGACGCGAAACTGGGTACTTACTATTCGTTAGTCTTGCCCTATCCGCT
ogt K-12 MG1655     ACGCTACAAACCAGACGCGAAACTGGGTACTTACTATTCGTTAGTCTTGCCCTATCCACT
                    *** ***************************************** *********** **
                                                     NarL I

ogt ETEC H10407     TATCTTTTTGGTGGTATGGCTGCTGATGTTGCTGGCGTGGTATCTTGTCGGTCTGCCGAT
ogt EAEC 042        TATCTTTTTGGTGGTATGGCTGCTGATGTTGCTGGCGTGGTATCTTGTCGGTCTGCCGAT
ogt EAEC 55989      TATCTTTTTGGTGGTATGGCTGCTGATGTTGCTGGCGTGGTATCTTGTTGGTCTGCCGAT
ogt BL21(DE3)       TATCTTTTTGGTGGTATGGCTGCTGATGTTGCTGGCGTGGTATCTTGTTGGTCTGCCGAT
ogt EHEC Sakai      TATCTTTTTGGTGGTATGGCTGCTGATGTTGCTGGCGTGGTATCTTGTGGGCCTGCCGAT
ogt K-12 MG1655     TATCTTTTTGGTGGTATGGCTGCTGATGTTGCTGGCGTGGTATCTTGTCGGTCTGCCGAT
                    ************************************************ ** ********
                        NarL II                               -10         +1

ogt ETEC H10407     AGGTCCGGGTATTTACCCACGTTTGTCTTAAGAGAGAACGGATGCTGAGATTACTTGAAG
ogt EAEC 042        AGGTCCGGGGATTTACCCACGTTTGTCTTAAGAGAGAACGGATGCTGAGATTACTTGAAG
ogt EAEC 55989      AGGTCCGGGGATTTACCCACGTTTGTCTTAAGAGAGAACGGATGCTGAGATTACTTGAAG
ogt BL21(DE3)       AGGTCCGGGGATTTCCCCACGTTTGTCTTAAGAGAGAACGGATGCTGAGATTACTTGAAG
ogt EHEC Sakai      AGGTCCGGGTATTTACCCACGTTTGTCTTAAGAGAGAACGGATGCTGAGATTACTTGAAG
ogt K-12 MG1655     AGGTCCGGGTATTTACCCACGTTTGTCTTAAGAGAGAACGGATGCTGAGATTACTTGAAG
                    ********* **** *********************************************
                                                              ogt start→
```

Supplementary Figure S4.

```
                                                                   TACYYMTnnAKRRGTA
ogt APEC O1          TATGCTTAAATACTGCGACAAACCAGACGCGAAACCTGGTACTTACTTTTTGTTAGTATT
ogt EPEC E2348/69    TATGCTTAAATACTGCGACAAACCAGACGCGAAACCTGGTACTTACTTTTTGTTAGTATT
ogt UPEC CFT073      TATGCTTAAATACTGCGACAAACCAGACGCGAAACCTGGTACTTACTTTTTGTTAGTATT
ogt K-12 MG1655      ------------ACGCTACAAACCAGACGCGAAACTGGGTACTTACTATTCGTTAGTCTT
                                   ** ******************  ********** ** ****** **
                                                                        NarL I

                                 TACYYMTnnAKRRGTA
ogt APEC O1          ------------AATCTTTTTGGTGGTATGGCTGCTGATGTTGCTGGCGTGGTATCTTGT
ogt EPEC E2348/69    ------------AATCTTTTTGGTGGTATGGCTGCTGATGTTGCTGGCGTGGTATCTTGT
ogt UPEC CFT073      ------------AATCTTTTTGGTGGTATGGCTGCTGATGTTGCTGGCGTGGTATCTTGT
ogt K-12 MG1655      GCCCTATCCACTTATCTTTTTGGTGGTATGGCTGCTGATGTTGCTGGCGTGGTATCTTGT
                                 ***********************************************
                                     NarL II                                   -10

ogt APEC O1          TGGCTTGCCGATAGGTCCGGGTATTTACCCACGTTTGTCTTAAGAGAGAACGGATGCTGA
ogt EPEC E2348/69    TGGCTTGCCGATAGGTCCGGGTATTTACCCACGTTTGTCTTAAGAGAGAACGGATGCTGA
ogt UPEC CFT073      TGGCTTGCCGACAGGTCCGGGTATTTACCCACGTTTGTCTTAAGAGAGAACGGATGCTGA
ogt K-12 MG1655      CGGTCTGCCGATAGGTCCGGGTATTTACCCACGTTTGTCTTAAGAGAGAACGGATGCTGA
                      **  ****** ************************************************
                        +1                                              ogt start→

ogt APEC O1          GATTACTTGAAGA
ogt EPEC E2348/69    GATTACTTGAAGA
ogt UPEC CFT073      GATTACTTGAAGA
ogt K-12 MG1655      GATTACTTGAAGA
                     *************
```

Supplementary Figure S5.

```
                                                             TACYYMTnnAKRRGTA
ogt STyphimurium      --AATTAACCGCCTCGCCAGGCTCTCTTTTTCTCCGACTACGATATTACTGTCGCTAAAT
ogt SCholeraesuis     --AATTAACCGCCTCGCCCGGCTCTCTTTTTCTCCGCCAACGATATTACTGTCGCTAAAT
ogt STyphi            --AATTAACCGTCTCGCCAGGCTCCCTTTTTCTCCGCCAACGATATTACTGTCGCTAAAT
ogt SDublin           --AATTAACCGCCTCACCCTGCTCCCTTTTTCTCCGCCAACGATATTACTGGCGCTAAAT
ogt SParatyphi        --AATTAACCGCCTCGCCAGGCTCCCTTTTTCTCCGCCAACGATATTACTGGCGCTAAAT
ogt K-12 MG1655       CAAACCAGACGCGAAACTGGG------------------TACTTACTATT--CGTTAGTC
                        **  *  **     *   *                      ** ** *  ** **
                                                                 NarL I

                                    TACYYMTnnAKRRGTA
ogt STyphimurium      GTGTTATCCCTGACTATCTTTTAAGGAGTATGGTTGCGGGTATTCCTGGCATGATATCTT
ogt SCholeraesuis     GTGTTATCCCTGACTATCTTTTAAGGAGTATGGTTGCGGGTATTCCTGGCATGATATCTT
ogt STyphi            GTGTTATCCCTGACTATCTTTTAAGGAGTATGGTTGCGGGTATTCCTGGCATGATATCTT
ogt SDublin           GTGTTATCCCTGACTATCTTTTAAGGAGTATGGTTGCGGGTATTCCTGGCATGATATCTT
ogt SParatyphi        GTGTTATCCCTGACTATCTTTTAAGGAGTATGGTTGCGGGTATTCCTGGCATGGTATCTT
ogt K-12 MG1655       TTGCCCTATCCACTTATCTTTTTGGTGGTATGGCTGCTGATGTTGCTGGCGTGGTATCTT
                       **   *  *    ********  *  ****** *** * * ** ***** ** ******
                                        NarL II                          -10

ogt STyphimurium      GTC--TCTTACGTTAGATTAAGACG-------ATGT---------GAGAGAACGGATGCT
ogt SCholeraesuis     GTC--TCTTACGTTAGATTAAGACG-------ATGT---------GAGAGAACGGATGCT
ogt STyphi            GTC--TCTTACGTTAGATTAAGACG-------ATGT---------GAGAGAACGGATGCT
ogt SDublin           GTC--TCTTACGTTAGGCTAAGACG-------ATGT---------GAGAGAACGGATGCT
ogt SParatyphi        GTC--TCTTACGTTAGATTAAGACG-------ATGT---------GAGAGAACGGATGCT
ogt K-12 MG1655       GTCGGTCTGCCGATAGGTCCGGGTATTTACCCACGTTTGTCTTAAGAGAGAACGGATGCT
                      ***  ***  ** ***     *          * **         ***************
                          +1                                                ogt start→
```
